# Meaning of Work Participation After Spinal Cord Injury in Bangladesh: A Qualitative Study in a Low- and Middle-Income Country Context

**DOI:** 10.1007/s10926-024-10244-5

**Published:** 2024-10-17

**Authors:** Mohammad Mosayed Ullah, Ellie Fossey, Rwth Stuckey

**Affiliations:** 1 Department of Occupational Therapy, Swinburne University of Technology, Melbourne, VIC 3122 Australia; 2 MedTechVIC Research Hub, Swinburne University of Technology, Melbourne, VIC 3122 Australia; 3 Department of Occupational Therapy, School of Primary Health Care, Monash University, Peninsula Campus, Frankston, VIC 3199 Australia; 4 Living With Disability Research Centre, School of Allied Health, College of Science, Health and Engineering, La Trobe University, Melbourne, VIC 3086 Australia; 5 Centre for Ergonomics and Human Factors, School of Psychology and Public Health, College of Science, Health and Engineering , La Trobe University, Melbourne, VIC 3086 Australia

**Keywords:** Work, Spinal cord injury, Disability, Lower-and-middle-income countries, Participation, Barrier and facilitators

## Abstract

**Purpose:**

This study aimed to explore the meaning of work participation for people with spinal cord injury (SCI) in Bangladesh.

**Methods:**

Narrative inquiry methodological framework was used to explore the meaning of work participation after SCI. Face-to-face interviews with twenty adults with SCI, who were either living in the community or in-patients at a rehabilitation center. The Worker Role Interview questionnaire was used as an interview guide. Participants were descriptively analyzed in two groups, rehabilitation participants and community participants. Their transcripts were analyzed using individual narrative analysis to understand the meaning of their experience at an individual level and then the findings from the individual narrative analyses were summarized using thematic analysis to identify themes that collectively represented the meaning of work after SCI in Bangladesh.

**Result:**

Five themes were identified from the interviews: “work life before injury”; “current life in relation to work”; “framing future prospects of work participation”; “motives for working”; and “enablers of work participation.”

**Conclusion:**

The meaning of work is subjective and is influenced by the participants’ pre-existing experiences and other factors related to their work life, such as work preferences, habits, and daily routines. Therefore, creating opportunities to better understand the meaning of work for each individual and incorporating these factors into rehabilitation are keys to sustainable rehabilitation outcomes.

## Introduction

Globally, the incidence of people with spinal cord injury (SCI) is high in young adulthood and peaks in those aged 30–34 and 50–54 years [1, 2]. It is common for people to be employed at the time of their injury. Hence, it is important that people with SCI are able to re-join the workforce to regain economic self-sufficiency, meaningful work roles and identity, and re-establish their place within community [3]. Yet, the rate of return to work after SCI globally varies from 5 to 40% [4, 5]. Many interrelated factors are known to impact successful work participation after spinal cord injury. These include personal factors, e.g., age, time since injury, severity of injury, education, self-management skills, societal attitudes, and living area or work environments; some of which are modifiable factors based on the country context. Few studies have been conducted in low-middle-income countries (LMIC) like Bangladesh, where income levels and limited rehabilitation following SCI may be expected to contribute to clear differences in outcomes [5–7]. Several studies in Bangladesh have investigated the return to paid employment of people with SCI [8–10]. Barriers identified included societal attitudes, misconceptions about disability, gaps between skills and available jobs, inaccessible work environments and transport systems, and limited financial supports [8, 9, 11]. Nahar et al. [10] identified a significant need for financial support to start economic reintegration following SCI, while Hossain et al. [8] found loss of income is common, with 91% of those families impacted dropping below the poverty line after injury. However, none of these studies investigated the process of regaining work after SCI and how people with SCI in Bangladesh view their work prospects or make decisions about their future work. To guide rehabilitation practice in Bangladesh, it is important to further understand the meanings ascribed to work by individuals with SCI, whether positive, challenging, or neutral, and the significance attached to work for them. This understanding would assist local Occupational Therapists and other rehabilitation professionals to explore work meanings and preferences with patients and to support their decision-making about future work, thereby enhancing patient-centered SCI rehabilitation [3]. Therefore, this study aimed to explore the meaning of work participation for people with SCI in Bangladesh. In this study context, ‘work participation’ refers to participating in any activity/task that participants considered as their main worker role, paid or unpaid. It was intentionally not limited to paid employment so as to be inclusive of women’s work participation in household activities being, culturally, their main productive role within the family structure in Bangladesh [12].

## Methods

### Study Design

A narrative inquiry approach was used to explore the meaning of work participation after SCI in Bangladesh, through each patient’s life story pre- and post-SCI, from their own perspective [13]. This approach was chosen to facilitate understanding of individuals’ experience over time and in context [14]. Stories allow people to communicate and easily explain their experiences, current life, or major events. Their stories were not limited to personal information but also provided information about socio-cultural life, society, and their environment [15]. They also enable researchers to understand the meanings and interpretations that individuals ascribe to their choices and actions [16].

### Researcher Positionality and Roles

First author (MMU) is a Bangladeshi Occupational Therapist with 12 years of practice experience at the time of data collection as part of his PhD project. CRP had been the previous workplace of MMU, 2 years prior to the interviews. He had prior experience of working with SCI people. RS and EF are the academic supervisors and both have a background in occupational therapy, expertise in qualitative research, rehabilitation, and work-related research.

### Local Context

The study was conducted in Bangladesh through CRP, a non-government, not-for-profit organization. CRP is the only specialized rehabilitation center in Bangladesh. People are admitted to CRP from hospitals, and the community throughout Bangladesh to receive rehabilitation which includes inpatient and outpatient allied health services for people with SCI using a holistic medical, psychological, vocational, and economic approach [17]. All SCI participants in this study participated in a vocational rehabilitation program, which includes vocational assessment, training, job advocacy, and financial support.

### Participants and Recruitment

Purposive sampling was used to select individuals who met certain criteria and would provide information-rich cases [18]. The purposive sample included men and women with varied injury characteristics, work histories, and living situations to develop a rich description from the perspectives of people with SCI in Bangladesh. The selection criteria included sustained tetraplegia or paraplegia; aged 18 years or older; had worked pre-injury; registered as a patient with service access from CRP for rehabilitation/pre-discharge within the inpatient facility or living in a community; capable of providing informed participation consent; and willing to share their experiences in an interview.

Eligible participants were invited via posters on boards in the hospital and flyers available in the communal areas or phone calls by a research assistant. Those interested were contacted by the first author to confirm consent and schedule an interview. Twenty participants completed interviews, at which point common issues were emerging. For example, participants consistently talked about the meaning of their work pre-injury, their expectations of family, and community attitudes about disability. This indicated data saturation and therefore, the first author stopped recruiting participants at this point.

### Data Collection

A demographic information sheet and semi-structured interview questions guided the face-to-face interviews. The demographic information sheet was developed by the research authors, the questions designed to obtain information about sociodemographic and clinical characteristics of the participant population.

The semi-structured interviews were conducted using the Worker Role Interview (WRI) version 10 [19], which is based on the Model of Human Occupation (MOHO) [20]. The WRI was chosen because it is designed to provide a picture of the factors that support and interfere with the success in returning to work [21]. The WRI questions provide a semi-structured guide to explore an individual’s values, interests, personal perceptions of ability, roles, habits, and perceptions of environmental factors that influence work participation after injury [19, 22]. The interviewer (first author) received prior training and practice experience in using the WRI to facilitate rich conversations with each participant about the meaning of work from their perspective.

### Piloting

The WRI interview questions were translated into Bangla (official language of Bangladesh) by the first author and checked with by another Bangla speaking OT. Practice interviews in Bangla with one person with a disability and three allied health practitioners assisted to simplify language translation and improve conversation flow in local dialogue.

Participants were interviewed in their preferred convenient location, either in the community or in the rehabilitation center. Participants were offered the option to have a family member to accompany them so as to foster a safe interview environment. Interviews were conducted by the first author, who is familiar with the local context in Bangladesh. Interview recordings were transcribed verbatim by the research assistant and first author.

### Data Analysis

Data were analyzed at the group level guided by steps of individual narrative analysis described by Gibbs, cited in Liamputtong [23], and thematically using the steps described by Braun and Clarke cited in Liamputtong [23].

All three authors independently extracted the raw coding from two participants' interview transcripts and met face-to-face to discuss the similarities and differences. Following this discussion, MMU completed the raw coding of the remaining interview transcripts. MMU and RS used these raw codes to develop a narrative story following the sequence of significant events chronologically at the individual level of each participant. Each participant’s story was analyzed using narrative analysis to understand the meaning of their experience at an individual level.

The findings from the individual narrative analyses of all twenty stories were then summarized using thematic analysis to identify themes that collectively represented the meaning of work after SCI in Bangladesh. MMU and RS independently revisited all the raw coding and participants’ quotes to develop categories. After combining all the categories, preliminary themes were developed. The overall themes were improved by renaming or merging some categories to enhance their coherence. At each step of the analysis, the authors regularly discussed similarities and differences until they reached a consensus.

Previous studies have used this approach of combining narrative analysis and thematic analysis for people with SCI [24, 25].

### Reflexivity

During the data collection, following each interview, the researcher (MMU) recorded technical decisions, understandings of prior assumptions, beliefs, and challenges with participants in a reflective journal. The research team critically reflected on their own views and beliefs in relation to the findings through regular open discussion during the analysis and manuscript development phases and the writing of personal reflection notes. Findings used verbatim quotations to support the interpretation.

## Findings

Twenty adults with SCI aged 19–59 years participated. Table 1 summarizes the sociodemographic and clinical characteristics of participants recruited as in-patients from the rehabilitation center (RP) (*n* = 10) and from the community (CP) (*n* = 10).

**Table 1:** Sociodemographic and clinical characteristics of participants

Demographic variables	Setting* n* (% of all participants)		Total *N*
	Rehabilitation center	Community	
*Age range (years)*			
18–29	5 (25%)	4 (20%)	9
30–44	3 (15%)	3 (15%)	6
45–60	2 (10%)	3 (15%)	5
*Gender*			
Male	8 (40%)	8 (40%)	16
Female	2 (10%)	2 (10%)	4
*Marital status*			
Married	8 (40%)	4 (20%)	12
Separated/divorced after injury	1 (5%)	2 (10%)	3
Unmarried	1 (5%)	4 (20%)	5
*Educational background*			
No education	1 (5%)	2 (10%)	3
Primary	3 (15%)	2 (10%)	5
Junior secondary	4 (20%)	3 (15%)	7
Secondary	1 (5%)	1 (5%)	2
Higher secondary or above	1 (5%)	2 (10%)	3
*Primary previous occupation*			
Manual labour	3 (15%)	5 (25%)	8
Small business—self-employed	3 (15%)	2 (10%)	5
Office job	2 (10%)	1 (5%)	3
Home-making	1 (5%)	1 (5%)	2
Paid domestic work	–	1 (5%)	1
Driver	1 (5%)	–	1
*Home region type*			
Rural	6 (30%)	8 (40%)	14
Urban	4 (20%)	2 (10%)	6
*Family size*			
1–4 members	6 (30%)	6 (30%)	12
5–8 members	4 (20%)	4 (20%)	8
*Type and level of injury*			
Paraplegia complete	4 (20%)	3 (15%)	7
Paraplegia incomplete	2 (10%)	3 (15%)	5
Tetraplegic complete	3 (15%)	4 (20%)	7
Tetraplegia incomplete	1 (5%)	0	1

Overall, the characteristics of both groups are similar although fewer in the community group were married. Most participants contributed to their households financially pre-injury; two females contributed as both the primary income source as well as undertaking domestic work. Most participants were relatively young at the time of injury (mean 32 years, SD ± 5). Injuries were from falling, such as from a tree during fruit-picking season or from a building during construction work, the majority occurring at their workplace (40%) or in road traffic incidents (30%). RPs had been injured 4–15 months prior to interview (mean 8 months) and CPs between 1 and 17.5 years (mean = 8.25 years) prior. Many participants reported continuing health complications associated with SCI, including muscle spasms (*n* = 15), burning sensations (*n* = 13), pain and postural hypo/hypertension (*n* = 5), and pressure sores (*n* = 6). Eighteen participants including all CPs were using manual wheelchairs for mobility. One of the RPs used a walking frame and another with tetraplegia, a ‘long-trolley’ (trolley modified with big wheels under a bed).

### Themes Related to Meaning of Work After SCI in Bangladesh

Overall, the findings suggest that participants’ previous work experience influenced the process toward their post-injury work participation. Steps toward work participation were positively influenced by the individual’s anticipation and thinking about working; their motives for working; and existing contextual enablers. Participants were not able to participate in work when challenges impacted to the point where they overrode these three positive influences.

**Fig. 1:**
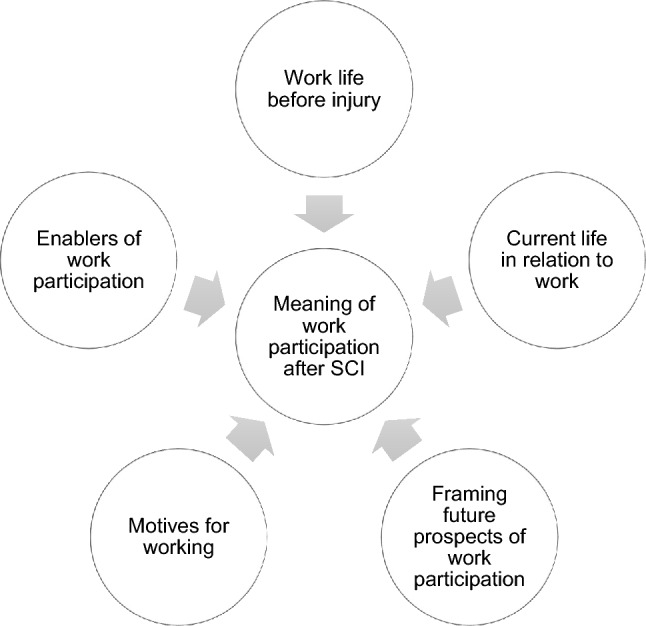
Themes related to the meaning of work participation after SCI in Bangladesh

The qualitative analysis generated five themes that describe the narrative development of the meaning of work participation after SCI in Bangladesh, as summarized in Fig. 1. Each theme is presented with sub-themes with illustrative quotes from participants’ interviews, using pseudonyms to protect their identities.

### Work Life Before Injury

All participants had experiences of working before injury and had developed views about their work that defined their identity, both at and outside work. These meanings included managing financial needs and/or supporting their family; connecting with others beyond home; and work as a source of feeling good and satisfied with their own abilities.

#### Work as a Means of Managing Financial Needs and/or Supporting Their Family

Pre-injury, most participants were financially contributing family members and the only income-earning family member. Their work had meaning both as a source of income and satisfaction with being able to improve the lives of spouses, children, or elderly parents:

“Pulling the rickshaw was a hard job. However, in the end it gave me a good feeling. When I counted the money, the memory of the struggle faded away. … I had sufficient income from that work. So, I could ensure a better life for my children. We also had good food, and we were able to lead a healthy life.” (Chonchol, RP, rickshaw puller).

#### Work as a Means of Connecting with Others Beyond Home

Opportunities for meeting others, communicating and working together were aspects of work participants particularly enjoyed. Regardless of the complexity and intensity of work, in factories or outdoors, participants described valuing contact with people from different backgrounds, sharing views, and workplace companionship:

“We worked in a team. I never felt an outsider. We always had sweet moments full of laughter. Though people from different districts worked there, they were connected by their shared views and opinions.” (Oshim, CP, construction worker).

#### Work as a Source of Feeling Good and Satisfied with Their Own Abilities

Working pre-injury brought feelings of pleasure from facing challenges and overcoming obstacles, satisfaction with completing assigned tasks, and gaining the capacity to help others. As Joynal, RP, Manual Labour, noted, while “work brings money, there is another factor of satisfaction. So, there is no benefit without work in life.” Most described their pre-injury work as physically demanding, but also rewarding:

“I enjoyed agricultural work … You feel good if there is enough rice in the field. When you have got more rice to carry, you never feel tired of this.” (Gias, CP, farmer).

### Current Life in Relation to Work

RPs and CPs described their present lives differently in relation to work. Most RPs were occupied in structured daily programs, whereas those in the community described more varied work experiences.

#### Contrasting Life Before and After Injury

CPs often described their current life as ‘*a completely different situation,’* emphasizing their current restricted involvement compared to previous participation, e.g., earning a living, getting out, and connecting with others. They spoke of missing their prior working life, feeling lonely, and sad about staying at home:

“I had everything. I enjoyed the independence after work. I spent earnings from my income on whatever I chose to. … What I do all the day now is keep sitting or just sleep. That’s how I pass all the day.” (Kona, CP, paid maid, and home-maker).

Participants felt these losses socially too; not being at work meant being alone or “*imprisoned*” at home, unable to meet others and finding their friendships reduced:

“I am not able to go out. I feel lonely and quiet within myself. No, I do not share with anyone. Only people like me can understand me, no one else. So, I am not interested in talking with others. No one is even interested in knowing this. Since the injury my number of friends has decreased. … Society also sees me negatively, as I am not at work. At this moment I cannot share my sad feelings with others”* (Samsul, CP, small business owner).*

Further, loss of physical control added to these feelings and the difficulties of facing people and work:

“My bowel and bladder have no control, that’s why I cannot go outside to meet with people, I feel bad for that. If I wish visit anywhere I cannot do that. If I can sense the bowel and bladder movement, and I could ask someone to help me…… That’s why I do not feel like working, I feel shy and shameful.” *(Gias, CP, farmer).*

Importantly, having lost work and their primary family (income, home-making, child-rearing) and social roles, participants without work described feeling impoverished, sad, empty, or being of less value, all being hard to bear.

“Yes, I feel upset. I feel upset because I worked before. I had a beautiful life full of enjoyment. But, what now? There is no pleasure in poverty. Necessity knows no law.” (Sonia, RP, garment maker).

Contrasting with the sense of loss expressed by non-working participants, those in work described with pride, being able to connect with others and contribute to their lives:

“I mostly enjoy the work which I can do while travelling. When I go to Savar, being behind the rickshaw for work, I can talk to 10 or more people. I can make them understand my situation. I like this very much.” (Emon, CP, manual labour).

In comparison, RPs kept busy in the rehabilitation centre’s structured daily programs found the regime was a source of hope in relation to work:

“When I came to CRP (name of a rehabilitation centre), I thought from seeing other people (with wheelchairs) that as they can do it, so surely, I will too. I might have to sacrifice something. I have to work now with a wheelchair instead of my legs.” (Akhand, RP, manual labour).

### Framing Future Prospects of Work Participation

Regardless of their current working status, all participants expressed a desire to work, each individually, framing their situation and future work. Most anticipated support from their families and communities, basing their ideas and strategies for future work participation on that support. Whether or not these expectations were likely to be realized, anticipating and thinking about work seemed an important stepping-stone toward work participation after SCI.

#### Ideas, Uncertainties, and Planning Strategies for Work

RPs described preparing for their future working life, taking part in skill development activities, volunteering, and applying for jobs. They also described preparing themselves mentally for work by considering what work might be suitable and how to manage their limitations and physical environments. Most expressed interest in shopkeeping activities, possibly influenced by the rehabilitation program culture, but also based on their perception that this work would be within their physical capacity:

“My previous kind of work is not possible in future. I have a plan to work till my last breath. I will sell home-made food. I have the idea of the set-up, of a table and a burner.” (Dewan, RP, small business owner).

Female participants expressed deliberations considering managing a shop and tailoring as work that would suit their capacities:

“Although I need another person to help me, I could feel the joy of independent work and earnings from the shop.” (Kona, CP, paid maid & home-maker).

Nevertheless, not knowing one’s capacity for work tasks introduced uncertainty in planning for future work and challenged confidence in their work possibilities:

“Yes, I will go back to my previous work. But I am not sure how much I can! Because my function will be slower, and I am slower! So, I don’t know if I can continue to work.” (Tomal, RP, software engineer).

#### Anticipating Support from Family

In their future work plans, most RPs were assuming that family members would be their assistants. Notably, male participants spoke of expecting the women in their families to not only undertake domestic and caring roles but to also assist in their work:

“I am thinking about running a shop like a tea stall with the help of my wife, and that’s why I have taken vocational training too. As I don’t have enough strength in my hands or legs, I have to use a wheelchair for the work. I can supervise and provide verbal instructions.” (Chonchol, RP, rickshaw puller).

In contrast, female participants commonly emphasized not relying on family members to support their participation in work:

“It’s my responsibility to take care of my child. I cannot just delegate it to someone else. I feel the same responsibility as everyone else in the family.” (Niru, CP, home-maker).

Consistent with the aspirations of RPs, most working CPs described the value of family members actively helping them run their business, often involving challenging stereotypical norms.

“If my wife is confident to do things, I have the capacity to be doing this … as a woman, she did not understand the business, did not recognise the weights scales, was not able to count money, did not understand anything. Then after teaching her, and by explaining, explaining, and explaining I made her understand and kept the business running. She also dared to do this. She saw there is no other way. She bravely said, “yes, I will be able to do it.” (Wasim, CP, small business owner).

#### Anticipating Support from the Local Community

Some participants were expecting to start working after returning home with community supports, such as local organizations focused on the initial set-up for a business:

“The local people are very helpful. They helped me a lot, still doing so, and will continue it in future too. The locals regret and feel sad for me and wish for me to go back to them … but I don’t have the capital. But, if {rehabilitation centre} and the local people help me, I could continue till I die, Insha’Allah. I hope I will.” (Akhand, RP, manual labour).

#### Accepting the Situation

Currently working participants also spoke of viewing themselves as not being limited by their disability and functioning in a manner no different from that of a healthy person:

“I don’t think of myself as a cripple. I do the things a normal person can do. I don’t think my son is deprived of anything as his mother is a wheelchair user.” (Niru, CP, home-maker).

Some participants also emphasized their efforts to focus on living, their current working status, and their future, rather than on their illness:

“I think I am fully adapted to my new life now. I had to go through a lot of pain and trouble. But, I believe there is no use overthinking about the past; rather it’s high time to act for the future. (Oshim, CP, construction worker)”

### Motives for Working

Participants expressed varied motives for working, providing an understanding of the importance of work in their lives after injury related to family, dignity, feeling healthy, connecting with, and inspiring others.

#### Fulfilling Family Responsibilities

Family responsibilities was a strong motivation for both male and female participants to work, aligned with their sense of obligation to contribute financially and/or through home-making activities, as they did previously, in keeping with Bangladeshi social norms. Similarly, wishing to provide for their children’s future was a strong motive for working among participants with children:

“I have the responsibility for my son’s future. … I also need to take care of the role of landlord for this five-storeyed building as I am the elder son of my family, like the collection of rent, maintenance, dealing with contractors for modifications or plumbing.” (Polash, CP, office job).

Financial motivations to work also concerned repayment of debt related to treatment and lack of other support within the family:

“We had to take a loan during the treatment, in 2001, and now I am paying it back slowly.” (Wasim, CP, small business owner).

#### Living a Life with Dignity

Participants were interested in work as a means of being independent and establishing their identity and pride. The dignity of work assisted overcoming the sense of being a burden on family following SCI, with personal pride in working themselves, rather than begging:

“Many people appreciated me and said that “the way you are living now, you are doing better than previously, because many people with wheelchairs live by begging, but you set up a shop instead of begging”. I feel good to hear them say this.” (Habib, CP, manual labour).

#### Feeling Healthy

While SCI had dramatically changed participants’ physical and mental health and capacity, all had been very physically active pre-injury. Both RPs and CPs expressed their interest in working to keep themselves healthy, emphasizing both its physical and mental benefits.

“If I had no work, I might have pressure sores and other problems. But if I work, it will be like an attachment. I have to go to the shop and open it. So, first I need to stay clean. I would have a good mind if I could work. I would feel fresh and different.” (Bishwash, RP, small business owner).

The difference experienced in body and mind after being able to work was clearly expressed:

“My mind was not in a good state when I was at home and was doing nothing. I always felt imprisoned in the room. So, I got pressure sores from staying on bed all the time in a room … Now, I wash the clothes, sweep the floor, and clean the room which seems like the exercise to me. I cut the fish, vegetables and paste the spices which is exercise too for me.” (Niru, CP, home-maker).

Working was appreciated as occupying participants in their daily life and distracting them from their disability:

“When I am at work, my thoughts are with work, not thinking about my disability. For this reason, work is also important to me.” (Niloy, CP, manual labour).

#### Connecting with the Wider World

Similarly to before injury, after injury most participants described connections with other people and the wider world through work as important motives, beyond economic ones, for working:

“If I am inside a home one or two people might come. But if I am at my shop everyone in the world is close to me. They are coming to the shop and I am chatting with them. Then I can hear about the news about home and abroad.” (Wasim, CP, small business owner).

#### Inspiring Persons with Disability

Several participants had chosen or were seeking work through which to become role models, to create networks with other individuals with disabilities, and to inspire them about possibilities for work:

“I want to do social work for people. My husband will help me. I am well-known in the local area as I help people. If the local people with disabilities face any problems, I go to them. I think I am living a beautiful life, so why can’t they!” (Niru, CP, home-maker).

### Enablers of Work Participation

Every participant experienced challenges, from issues directly associated with their injuries to challenges in their physical and socio-cultural context. These challenges underscored the importance of enabling factors in their pursuit of work. CPs identified factors that enabled work participation, many of which were also anticipated by RCs for their future work participation.

#### Having a Positive Outlook, Attitude, and Determination

Participants with a positive outlook on life described their determination to engage in work despite changes in their health due to SCI, considering work was a ‘*milestone of success’* post-injury. This positive thinking was influenced by seeing other people with SCI working in the rehabilitation center and expressed in various ways, including commitment to protective health habits to enable working:

“I can continue working as long as I live. But, I wish not to have pressure sores. I must always stay clean to prevent these sores.” (Bishwash, RP, small business owner).

Participants also focused on strengths, such as personal qualities, they could draw on:

“I don’t think of myself as crippled because of my mentality, courage and attitude. I might not walk, but I travel alone by bus for nine hours and go to my parents, using the power of my mind.” (Niru, CP, home-maker).

At the same time, these positive outlooks took time to develop, with thoughts about their loss of ability, feeling left behind, fears of being alone and of falling and sadness, all noted as challenges.

“I was very demoralized right after injury …. I lost my inner drive and determination and got demoralised. … Ultimately I became very inactive, and I remained inside my home unless there was some very specific task. …Now everyone can see the benefits of me going out. If I go out, I am livelier.” (Polash, CP, office job).

#### Not Being Put Off by the Negative Attitude of Others

Developing an attitude of ‘*not being put off*’ in their effort to work by the attitudes of others was necessary for perseverance. Participants commonly described encountering negative attitudes regarding the cause of their injuries, disability, and expected roles as they sought to engage in work.

“If we try, we can work. They (villagers) think we cannot work at all. Even my family members feel the same. These attitudes create the problem.” (Niloy, CP, manual labour).

Expressed by family, friends, neighbors, and strangers, these negative attitudes often reflected lack of understanding and widely held stigmatizing views, and caused participants hurt or shame and sometimes for their families too. Participants in the city and in rural villages described being stared at in their wheelchairs, “*like a strange creature*” (Samsul, CP, small business owner), a source of embarrassment:

“So, when I go out, people treat me like they are in the zoo. They give me a look as if they are watching something totally new. … I feel a little ashamed and embarrassed that all these people are looking at me.” (Emon, CP, manual labour).

Participants spoke of needing to not let these views stop them, challenging these attitudes as ways to move ahead with pursuing work:

“Yes, many people say so many different things. However, it does not stop me. Nah {confidently}, I never get weakened by this.” (Wasim, CP, small business owner).

For some, negativity provided the impetus to prove they were capable of working and adding value in the community.

“I don’t think the negative words made me fall behind. I was hurt, so these words pushed me towards going ahead. I have focused on these words in a way that challenged me to prove them wrong.” (Emon, CP, manual labour).

#### Ability to Match Prior Knowledge and Skills with Work

Some participants chose their work based on their existing transferrable skills, like shopkeeping, sewing, computer engineering, and event management, planning future work based on prior knowledge including context and networks.

“Yes, there will be a demand for tailoring work. I have been living in {place} for a long time. So, everyone knows me around here. They also know that I worked as an operator in a garments factory. So, the quality of the work might not be bad. These are the reasons they will come.” (Sonia, RP, garment maker).

“I can mix with people at all different levels. This quality of mixing with people helps me a lot. This quality helped me to get work done, as I can easily ask for assistance with my requirements.” (Polash, CP, office job).

Similarly, existing social skills such as the ability to get along with different kinds of people were identified as useful for contributing to disability advocacy:

#### Receiving Support from Family, Friends, and Neighbors

Working participants highlighted the significance of receiving support from family members, friends, or relatives toward overcoming physical and psychological challenges and social and financial barriers.

“Though I am sick, after seeing her {wife’s care and support}, I become braver. Like family is caring for me so much, so let me do something for them to give in return by being brave. She goes to hut, bazaar, market, everywhere, meaning I am walking by her legs, talking by her mouth, but with my brain.” (Wasim, CP, small business owner).

For women, such as Niru, whose parents assisted in setting up and financing a local shop, receiving her parents’ support held additional significance given the negative attitudes expressed toward her both as “*a crippled person*” and a woman operating a shop in the local market:

“My parents used to tell me that “you don’t need to pay heed whatever people say. Your target is to move forward” … Yes, many used to say that it looks wrong that I am continuing to have a shop in the market because I am a woman.” (Niru, CP, home-maker).

Community support, (visits from friends, assistance with treatment costs, and others’ prayers), also gave participants hope and boosted their confidence to start a new work life, in the face of potential others’ misunderstanding of their working capacity:

“Yes, there are good people too. I have got friends, and good neighbours. They come to help me regularly. I have got four or five friends who visit me regularly.” (Samsul, CP, small business owner).

#### Exploring Strategies to Manage Physical Barriers

Exploring practical strategies for managing physical environmental issues related to possible work was important. RPs described strategies necessary to enable them to manage future work participation, including workplace modifications:

“The goods must be kept within my reach. I also need to be able to rest to continue shopkeeping. I wish to make a room beside the shop where I can perform my catheterisation.” (Akhand, RP, manual labour).

Being competent in wheelchair use to overcome challenges is important, wheelchairs being crucial to participating in life, as Niru described:

“When I transfer to bed and the wheelchair moves away, I feel something is moving away from my life. I don’t see anything as an obstacle if the wheelchair is there with me.” (Niru, CP, home-maker).

## Discussion

This study aimed to explore the meaning of work participation after SCI in Bangladesh. This study found that pre-injury meaning of work influenced participant expectations related to future work prospects. Prior to injury work created opportunities to engage in meaningful occupational participation through performing roles required to support their families and connecting with social networks. More importantly, work was their source of identity and satisfaction, an important consideration when exploring motivation for future work. This finding is consistent with recent research into work disability from chronic musculoskeletal injury which suggests that choices for new directions are firmly based on previous work meanings [26]. Similarly, when considering ‘work participation,’ this study and previous research found that patients with SCI undergo a continuous process of comparing and contrasting previous work life with their current situation [27].

The experience of participants grieving their current life and their loss of work capacity is common. Post-injury, most participants compared their current with their previous life as a standard against which they measured their abilities and expectations. The motives for work after SCI included feeling healthy, living with dignity, connecting with the external world, and fulfilling family responsibilities, consistent with factors previously found to influence sustaining work in the longer term, along with personal factors such as attitude, and ability to adapt, and self-advocate [28, 29]. An individual’s psychological factors including positive attitudes, determination, and not being put off by others, supportive family, and extended network support have also been identified by others as key factors in sustaining a work role [28]. Our study also found having a busy daily life work schedule during the rehabilitation phase was useful, consistent with findings of Wilbanks and Ivankova [30] who identified the importance of early training and extended time in rehabilitation programs as preparation for work.

The wheelchair represented freedom and power for some participants, similar to findings in earlier studies of people with multiple sclerosis [31, 32]. In these studies, as in ours, the wheelchair was identified as having a positive impact on quality of life. Our study has illustrated that those people who are active in pursuing work highlight the value of accepting a new way of living life with SCI, anticipating support from immediate family members and their local community for their future transition to work. This support assists by suggesting ideas, dealing with uncertainty, planning strategies, and helping promote adaptability, all facilitatory factors for future work participation [33].

Different gender-based expectations of roles were identified: male participants expected women in their families to undertake both domestic and caring roles and assist them to work, while female participants emphasized their determination to not rely on others. Similar findings were described in previous research involving a lower limb amputee cohort in Bangladesh [34], where, when a male lost a limb, their female partner assumed many responsibilities, including paid work, but when a female lost a limb this work role reversal did not occur. While these studies [34, 35] were both in the Bangladeshi context, they may also reflect broader attitudes in many countries toward expectations of women, including women with a disability and work and carer roles.

### Strength and Limitation

Participants in this study were recruited from one specialized rehabilitation center service in Bangladesh. Therefore, the findings may have limited transferability to the experience of people with SCI beyond this organization. For practical reasons, it was also not possible to interview the same participant twice or conduct member checking. To address this issue, 20 persons with SCI were interviewed to enhance the range and quality of information gathered. There were more limited findings about home-making, compared to male-based tasks, due to fewer female participants with SCI being recruited. However, this gender bias toward male participants accurately reflects the Bangladeshi SCI patient population [36, 37]. The language used in interviews was Bangla, and transcripts were translated into English for analysis. This process was carefully executed and was time-consuming; however, there might remain a chance that the exact meaning of participants’ quotes was misinterpreted or the meanings distorted in translation.

### Implication for Practice and Future Research

The meaning of work is subjective and influenced by the participants’ pre-existing experiences and other factors related to work life. Attending to factors, such as work preferences, habits, and daily routines in rehabilitation helps to understand what a patient is interested in doing, and why and how. Understanding the ‘how to do’ aspect of the meaning of work in SCI rehabilitation is important when offering choices of work and developing and executing intervention plans to facilitate patients’ self-evaluation and long-term work participation.

Future research needs to focus on developing a context-specific service delivery model that integrates exploring the meaning of work following SCI from the early stage of rehabilitation and how it impacts obtaining and sustaining a worker role in post-SCI community life. Further research should include the local rehabilitation professionals’ view and means of exploring the meaning of work participation for patients with SCI.

## Conclusion

People with SCI expressed that the meaning of work participation develops through a complex interaction of personal and environmental factors. Besides physical injury, perceived challenges and enablers play a role in the likelihood of work participation. It is important to explore the meaning of work participation following SCI within the rehabilitation process to facilitate framing the prospect of work and its sustainability.

## Data Availability

No datasets were generated or analyzed during the current study.
